# Dietary Lactoferrin Alleviates Age-Related Lacrimal Gland Dysfunction in Mice

**DOI:** 10.1371/journal.pone.0033148

**Published:** 2012-03-27

**Authors:** Motoko Kawashima, Tetsuya Kawakita, Takaaki Inaba, Naoko Okada, Masataka Ito, Shigeto Shimmura, Mitsuhiro Watanabe, Ken Shinmura, Kazuo Tsubota

**Affiliations:** 1 Department of Ophthalmology, Keio University School of Medicine, Tokyo, Japan; 2 Division of Geriatric Medicine, Department of Internal Medicine, Keio University School of Medicine, Tokyo, Japan; 3 Department of Internal Medicine, Keio University School of Medicine, Tokyo, Japan; 4 Department of Ophthalmology, National Defense Medical College, Saitama, Japan; Washington University School of Medicine, United States of America

## Abstract

**Background:**

Decrease in lacrimal gland secretory function is related to age-induced dry eye disease. Lactoferrin, the main glycoprotein component of tears, has multiple functions, including anti-inflammatory effects and the promotion of cell growth. We investigated how oral administration of lactoferrin affects age-related lacrimal dysfunction.

**Methods and Findings:**

Twelve-month-old male C57BL/6Cr Slc mice were randomly divided into a control fed group and an oral lactoferrin treatment group. Tear function was measured at a 6-month time-point. After euthanasia, the lacrimal glands were subjected to histological examination with 8-hydroxy-2′-deoxyguanosine (8-OHdG) antibodies, and serum concentrations of 8-OHdG and hexanoyl-lysine adduct (HEL) were evaluated. Additionally, monocyte chemotactic protein-1(MCP-1) and tumor necrosis factor-α (TNF-α) gene expression levels were determined by real-time PCR. The volume of tear secretion was significantly larger in the treated group than in the control. Lactoferrin administration reduced inflammatory cell infiltration and the MCP-1 and TNF-α expression levels. Serum concentrations of 8-OHdG and HEL in the lactoferrin group were lower than those in the control group and were associated with attenuated 8-OHdG immunostaining of the lacrimal glands.

**Conclusion:**

Oral lactoferrin administration preserves lacrimal gland function in aged mice by attenuating oxidative damage and suppressing subsequent gland inflammation.

## Introduction

Lactoferrin (LF) is an iron-binding glycoprotein present in serum and exocrine secretions [1]–[3]. LF suppresses inflammation, promotes cell growth and DNA synthesis, and exhibits anti-angiogenic and anti-tumorigenic properties [1]–[6]. Moreover, various physiological qualities, such as anti-oxidative and anti-carcinogenic bioactivities, have been attributed to LF [1]–[3], [7].

Oxidative stress initiates a lipid peroxidation chain reaction, thereby disrupting membranes and organelles, as well as causing protein degradation, DNA breakage, and generating mutagenic lesions such as 8-hydroxy-2′-deoxyguanosine (8-OHdG) [8]–[10], which is a reliable marker of reactive oxygen species (ROS)-induced DNA modification. The resulting oxidative damage could contribute to the pathologic processes in various diseases, including cancer. In a recent study, Tsubota et al. demonstrated that bovine LF inhibits liver mitochondrial 8-OHdG formation in Long-Evans Cinnamon rats [6].

Tear LF levels have been reported to be an indicator of lacrimal secretory function [11]. LF concentration in tears has been reported to decrease in dry eye patients [11], [12]. Tear secretion decrease gradually over the age of 40 [13], and LF concentration is also decreasing with age [13], [14] and tear LF originates from lacrimal gland acinar cells[15], [16]. Therefore, it is reasonable that a LF decrease would have some relation to lacrimal gland function.

While Shimmura et al. reported a protective effect of LF against oxidative cellular damage in cultured corneal epithelial cells [17], our previous study revealed a correlation between lacrimal gland secretory function and age-induced dry eye disease in rats, which may stem from oxidative stress [18]. Although the in vivo metabolism of LF remains unclear, these findings led us to hypothesize that LF administration may result in a protective effect on lacrimal glands.

Therefore, we investigated whether orally administered LF is effective in preventing lacrimal dysfunction and whether this effect is related to the antioxidative properties of LF.

## Materials and Methods

### Experimental protocol

All procedures undertaken in the present study conformed to the principles outlined in the *Guide for the Care and Use of Laboratory Animals* published by the USA National Institutes of Health (NIH Publication No. 85-23, revised 1996) and were approved by the Institutional Animal Care and Use Committee of Keio University School of Medicine (permission No. 08067). All procedures were performed according to the ARVO statement for the Use of Animals in Ophthalmic and Vision Research.

Ten-month-old male C57BL/6Cr Slc mice were obtained from Tokyo Metropolitan Institute of Gerontology (Tokyo, Japan). The mice were housed in individual cages and fed ad libitum with powdered AIN-93G (Oriental Yeast Co., Tokyo, Japan). After a 2-month weaning period, 1-year-old mice were randomly divided into 2 groups. The control group (CTL) continued to be fed with the control diet (powdered AIN-93G) for the next 6 months, while the LF group was fed with the experimental diet formulated by adding 2% LF to the control diet (2% enteric-coatedbovine LF in food weight; NRL Pharma, Kawasaki, Japan). We determined the dose of 2% and duration of6month, following with the previous reports [6], [19], [20]. The animals were maintained on a 12-h light/dark cycle in an atmosphere-controlled room. Body weight of all individuals was recorded weekly. Total food intake was also recorded every week, and the daily food intake was calculated. Lacrimal gland weights were measured at 6 months, and serum samples were collected for biochemical analysis by auto-mated analyzer (HITACHI 7180 Clinical Analyzer).

### Tear secretion test (cotton thread test)

A phenol red-impregnated thread was placed on the temporal side of the lower eyelid margin for 30 seconds. The length of the moistened fragment was measured.

### Sample preparation

Mice were dissected after anesthetization. The lacrimal glands and conjunctiva were quickly removed under refrigeration (4°C). For real-time PCR, samples were immediately frozen in liquid nitrogen and kept at −80°C until tested. For histopathological analysis, samples were immediately embedded in an optimal cutting temperature compound (Tissue-Tek; Miles Inc., Elkhart, IN) and kept at −80°C until analysis.

### Histopathological examination

The lacrimal gland specimens were cut into 5-µm sections, stained with hematoxylin and eosin, and subsequently examined under a light microscope. In immunostaining for 8-hydroxy-2′-deoxyguanosine (8-OHdG), the specimens were blocked with normal horse serum; the sections were layered for diluted mouse anti-8-OHdG monoclonal antibody (Japan Institute for the Control of Aging [JaICA], Fukuroi, Japan). Antibody binding was detected with a horse anti-mouse IgG ABC kit (Vector Laboratories INC, Burlingame, CA) used according to the manufacturer's protocol, or sections were further treated with FITC-conjugated secondary antibodies (Jackson ImmunoResearch, West Grove, PA). Immunostaining for CD45, the specimens were incubated with anti-CD45 antibody (1∶100; eBioscience, San Diego, CA, USA). Samples were then treated with HRP-conjugated secondary antibodies (Molecular Probes, Carlsbad, CA). HRP-conjugated antibodies were visualized by the addition of diaminobenzidine tetrahydroxychloride. Nucleuses were counterstained with hematoxylin. These sections were examined under a light or fluorescence microscope.

### Transmission electron microscopy

A portion of lacrimal gland tissue was immediately fixed with 2.5% glutaraldehyde and subjected to the electron microscopic examination, as described previously [21]. One-micrometer-thick sections were stained with methylene blue, and the portions of interest were thin-sectioned and examined with an electron microscope (1200 EXII; JEOL, Tokyo, Japan).

### Measurement of 8-OHdG and HEL concentrations in serum

The levels of 8-OHdG and hexanoyl-lysine adduct (HEL) in the serum were measured with highly sensitive 8-OHdG Check ELISA and HEL ELISA (Japan Institute for the Control of Aging, Fukuroi, Japan) according to the manufacturer's protocol.

### Quantitative real-time polymerase chain reaction (PCR)

Total RNA was isolated using TRIzol (Invitrogen, Carlsbad, CA) according to the manufacturer's instructions and treated with DNase. RNA (1 µg) was used for reverse transcription (RT). Diluted cDNA (50×) was used for quantitative PCR (qPCR). The qPCR reactions were performed using the Light-Cycler System (Roche Applied Science, Penzberg, Germany) and the qPCR Supermix (Qiagen, Hilden, Germany) with primers (summarized in Table 1) for the amplification of monocyte chemotactic protein-1(MCP-1) and tumor necrosis factor-α (TNF-α) genes as inflammation markers, and the GAPDH housekeeping gene.

**Table 1:** Primers.

	sequence	bp
MCP-1	TGGTGATCCTCTTGTAGCTCTCC	23
	CCACTCACCTGCTGCTACTCAT	22
TNF-α	ATGAGAAGTTCCCAAATGGC	20
	CTCCACTTGGTGGTTTGCTA	20
GAPDH	TGTGTCCGTCGTGGATCTGA	20
	CCTGCTTCACCACCTTCTTGAT	22

### Statistical analysis

Statistical analysis was performed using a commercially available software package (Excel toukei, Social Survey Research Information Co., Ltd., Tokyo, Japan). The two-tailed Student's t-test was used for all analyses. Statistical significance was established at P<0.05.

## Results

### Body weight and serum parameters

No mice died throughout the experiment (total number of mice is 20). No significant difference in pre- and post-experimental body weight was observed between the CTL and the LF group (Table 2). In the biochemical analysis of the serum, unsaturated iron binding capacity (UIBC) and total iron binding capacity (TIBC), as well as the levels of non-esterified fatty acids (NEFA) and low density lipoprotein cholesterol (LDL-C) were significantly lower in the LF group than in the CTL group. Other biochemical parameters obtained are summarized in Table 3.

**Table 2:** Body weight and daily food intake.

	CTL group	LF group
Pre-experimental body weight, (average ± SD) (g)	43.6±2.9	42.9±3.6
Post-experimental body weight, (average ± SD) (g)	47.4±4.5	48.0±4.3
Daily food intake, (average ± SD) (g)	3.7±0.3	3.8±0.4

**Table 3:** Serum parameters.

	CTL group	LF group	P-value
TP (g/dL)	4.8±0.3	4.9±0.2	0.83
ALB (g/dL)	2.7±0.3	2.8±0.1	0.33
CRE (mg/dL)	0.3±0.3	0.4±0.6	0.46
Na (mEq/L)	0.3±0.3	0.4±0.6	0.46
K (mEq/L)	4.5±0.2	4.6±0.7	0.91
Cl (mEq/L)	112.6±3.3	114.0±1.2	0.16
Ca (mg/dL)	8.8±0.4	8.7±0.4	0.67
IP (mg/dL)	6.9±0.6	6.7±0.9	0.46
Mg (mg/dL)	2.9±0.2	2.7±0.1	0.2
Fe (µg/dL)	118.0±50.0	142.8±8.6	0.75
UIBC (µg/dL)	273.8±61.5	192.8±13.2	0.01*
TIBC (µg/dL)	391.8±36.8	335.6±17.7	0.02*
AST (IU/L)	147.6±63.9	111.2±49.2	0.46
ALT (IU/L)	124.4±72.0	61.0±27.1	0.29
LDH (IU/L)	882.0±130.9	650.0±367.2	0.46
AMY (IU/L)	2597.8±490.3	3181.8±382.3	0.07
T-CHO (mg/dL)	158.4±38.9	152.8±25.2	0.75
F-CHO (mg/dL)	36.6±8.2	37.4±6.0	0.91
E-CHO (mg/dL)	121.8±30.6	115.4±19.2	0.75
E/T (%)	76.8±0.8	75.6±0.5	0.03*
TG (mg/dL)	25.8±2.2	24.0±4.6	0.91
PL (mg/dL)	263.2±51.9	262.0±36.2	0.91
NEFA (µEq/L)	503.2±43.5	315.6±86.5	0.009**
LDL-C (mg/dL)	17.6±5.4	9.6±2.3	0.03*
HDL-C (mg/dL)	65.4±13.2	71.2±9.5	0.46
T-BIL (mg/dL)	0.1±0.0	0.1±0.0	0.45
TL (mg/dL)	390.6±88.5	380.4±59.2	0.75
GLU (mg/dL)	193.0±54.9	200.8±30.9	0.91

* p<0.05.

### Tear secretion

**Figure 1:**
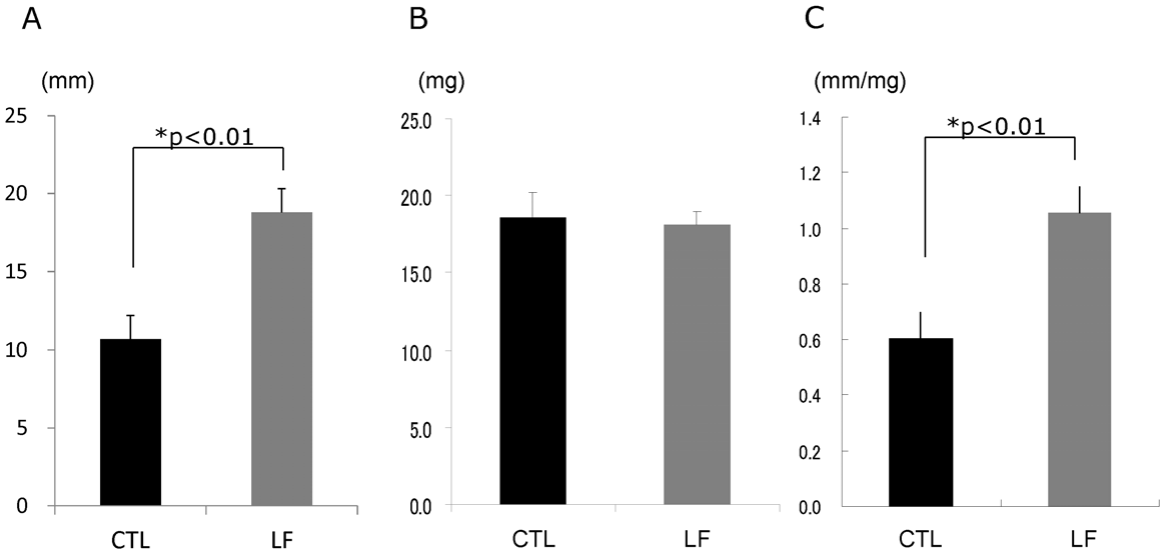
Tear secretion volume. A. Time course of the tear secretion. B. Lacrimal gland weight. C. Tear secretion corrected by lacrimal gland weight at 6 months. The volume of tear secretion was significantly larger in the lactoferrin (LF) group compared to the control (CTL) group. *p<0.01, N = 10, average ± standard error of mean).

At the 6-month time-point of the experiment, the tear volume measured by the cotton thread test was significantly larger in the LF group (average ± SD, 18.8±4.8 mm) than in the CTL group (average ± SD, 10.7±4.8 mm) (Figure 1A, p<0.01). No significant difference was found in the lacrimal gland weight (average ± SD, CTL: 18.6±5.0 mg, LF: 18.1±2.7 mg) between the 2 groups (Figure 1B). The tear secretion value corrected by lacrimal gland weight at the 6-month time-point was significantly larger in the LF group than in the CTL group (Figure 1C).

### Inflammatory changes and oxidative stress

**Figure 2:**
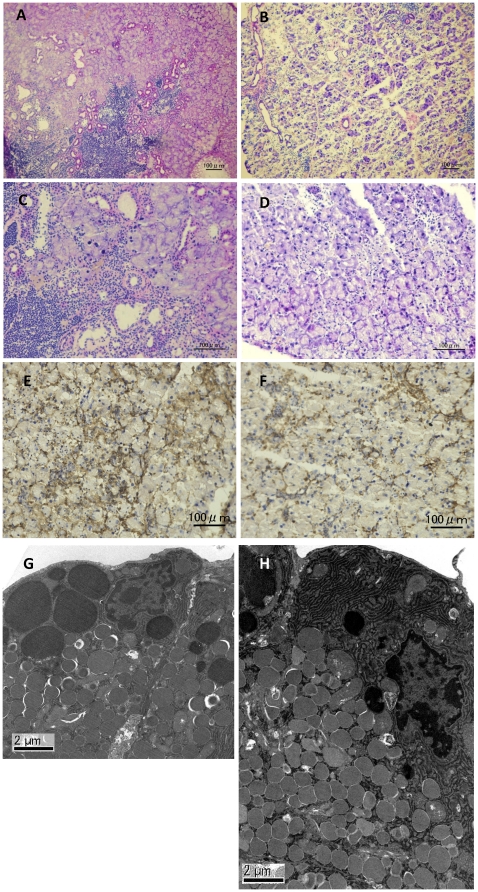
Morphological changes in lacrimal glands. Hematoxylin-eosin staining of control (A, C) and lactoferrin groups (B, D). CD45 immunostaining of lacrimal glands in the control group (E) and the lactoferrin group (F) Organelle morphology of control (G) and lactoferrin groups (H) examined by transmission electron microscope. Scale bar indicated 100 µm (A–F) and 2 µm (G, H).

**Figure 3:**
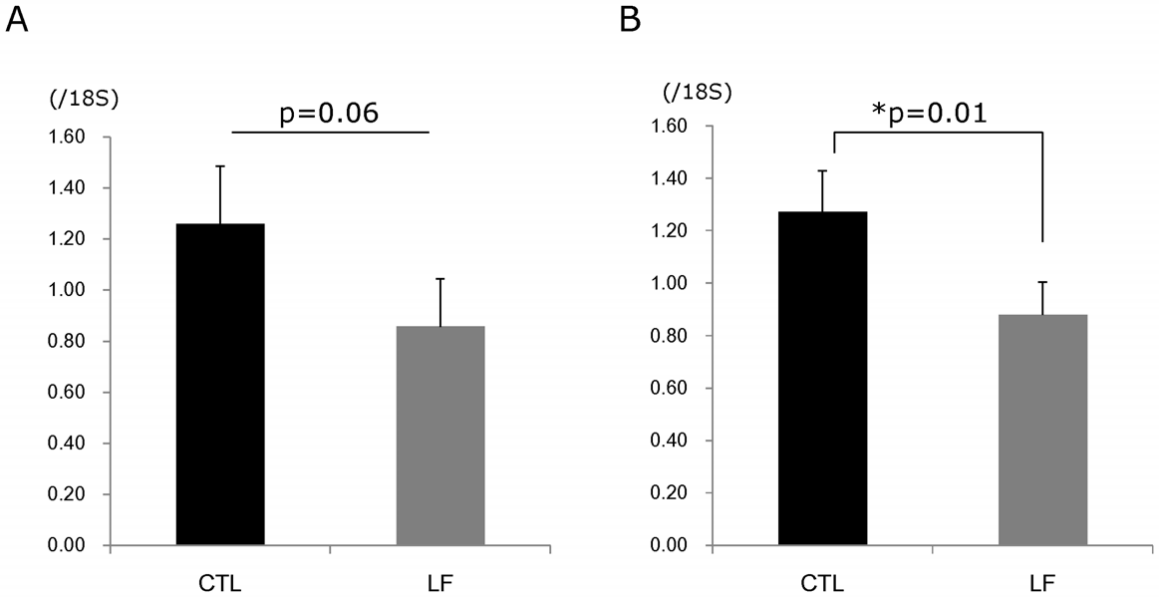
Gene expression analysis in lacrimal glands. A. Tumor necrosis factor-α (TNF-α). p = 0.06, N = 10, average ± standard error of mean (SE). B. Monocyte chemotactic protein-1 (MCP-1). *p = 0.01, N = 10, average ± SE. MCP-1 and TNF-α expression in the lactoferrin (LF) group were lower than the control (CTL) group.

**Figure 4:**
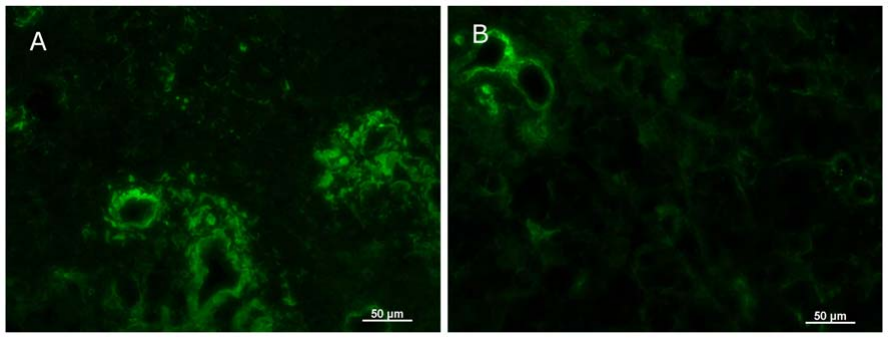
Oxidative stress marker in lacrimal glands. 8-OHdG immunostaining of lacrimal glands in the control group (A) and the lactoferrin group (B). Scale bar: 50 µm.

**Figure 5:**
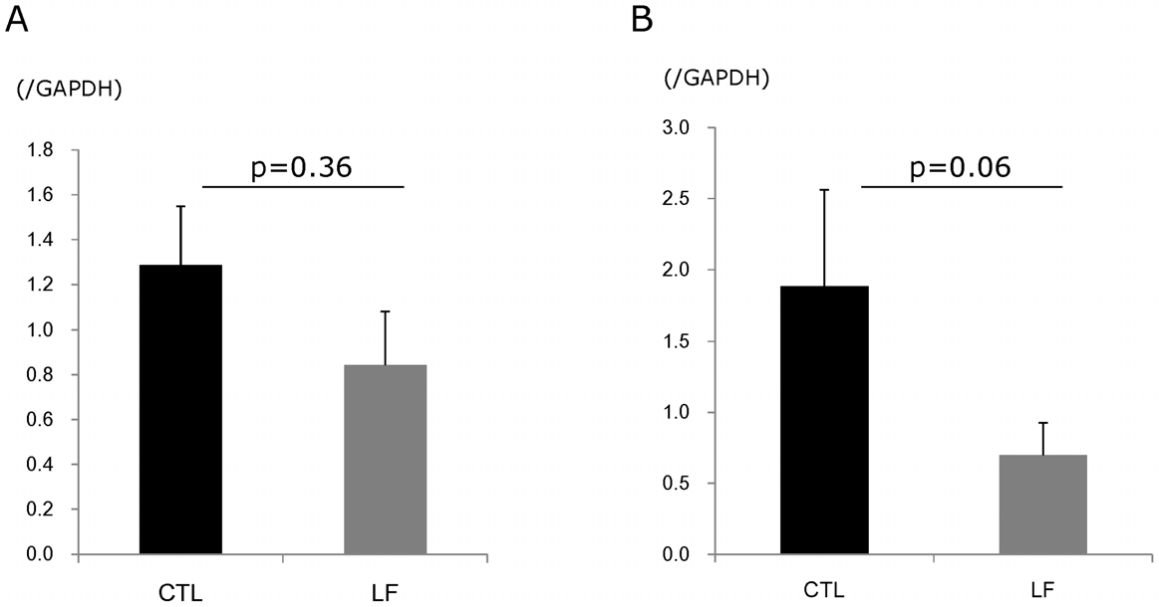
Gene expression analysis in conjunctivae. A. TNF-α. p = 0.36, N = 7, average ± standard error of mean (SE). B. MCP-1. p = 0.06, N = 7, average ± SE. MCP-1 and TNF-α expression in the lactoferrin (LF) group tended to belower than the control (CTL) group.

**Figure 6:**
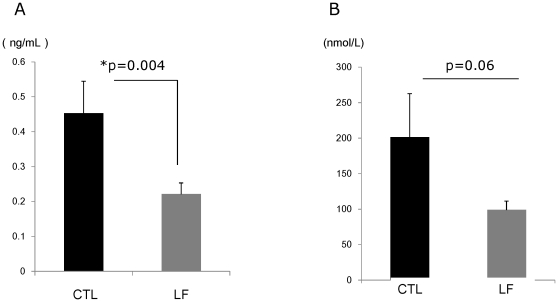
Oxidative stress markers in serum. A. 8-hydroxy-2′-deoxyguanosine (8-OHdG). *p = 0.004, N = 5, average ± standard error of mean (SE). B. Hexanoyl-lysine adduct (HEL). p = 0.06, N = 5, average ± SE. The concentration of 8-OHdG and HEL in the lactoferrin (LF) group were lower than the control (CTL) group.

To identify the morphological changes induced by LF in the lacrimal gland, histological assessment was performed. Hematoxylin and eosin staining of lacrimal glands showed that inflammatory cell infiltration was reduced in mice treated with LF, as compared to the CTL (Figure 2A–D). Immunostainings against CD45 also revealed weaker expression in the LF group than the CTL (Figure 2E and F). Electron microscopy of the acinar epithelial cells showed abnormal and disoriented vesicles in the control animal samples, whereas uniform vesicles were observed in the apical area of their LF counterparts (Figure 2G and H). Electron microscopy investigation also revealed that the rough endoplasmic reticulum architecture was better maintained in the LF group than the CTL (Figure 2C and D). Moreover, overall MCP-1 and TNF-α gene expression levels were lower in the LF experimental group (Figure 3). We next examined oxidative stress markers in the lacrimal glands. Immunohistochemistry of the lacrimal glands showed positive staining for 8-OHdG around the duct and acinar cells in the CTL group. In contrast, while the lacrimal gland tissues of the LF group showed positive staining in similar locations, the extent of the staining was markedly lesser (Figure 4). Subsequently, we applied our real-time PCR protocol to the conjunctiva samples in order to check the ocular surface status. MCP-1 and TNF-α expression levels tended to be lower in the LF group than in the CTL group (Figure 5) although no statistical significance was shown. We further performed ELISA assays for 8-OHdG and HEL in the serum. The concentration of 8-OHdG was significantly lower in the LF group than in the CTL group, and HEL concentrations showed the same tendency (Figure 6).

## Discussion

In this study, dietary LF seemed to provide an efficient treatment alternative to improve tear secretion in aged mice. Previously, Dogru et al. had demonstrated that oral administration of LF for 1 month improved tear stability and the ocular surface epithelium condition in dry eye patients with Sjögren syndrome [22]. This report clearly supports our results. However, while the previous study indicated the LF-induced increase in goblet cell density, we did not observe any differences in expression of goblet cell-related genes (mucin1, mucin4, mucin5AC, and SAM pointed domain containing ets transcription factor) between the control and the LF experimental group (data not shown). Further investigation of LF-containing eye drops is necessary to confirm their presumed goblet cell increasing effect [23], [24] and the consequent improvement in the ocular surface epithelium condition [17], [25], [26].

Dogru et al. revealed ocular surface condition improvement and tendency of increasing lacrimal gland secretion after 1month, but there was no significant difference. [22]. They included the severe Sjögren syndrome patients without symptomatic and objective improvement despite existing treatment, and their lacrimal glands would be damaged too severely to restore function by LF administration. When they continued to take oral LF, they might have improvement in tear secretion; we have detected a significant increase in tear volume after a 6-month intake of LF in mice. This may be partially explained by the difference in the intervention periods or the diversity of species. Furthermore, this study used aged mice, which suffered milder inflammation and less tissue damage than Sjögren syndrome patients. Because of the severity of tissue damage in Sjogren syndrome patients, LF reduced inflammation but couldn't improve tear secretion in 1 month. However there is a possibility that 6month administration could increase the tear volume for mice experimented upon in this study. Taken together, both human and mice experiments revealed LF supplementation was safe and effective therapy for lacrimal gland dysfunction, so we would use LF to add to the existing treatment for Sjögren syndrome patients or as a preventative the age-related decline of lacrimal gland function.

LF receptors are present in human nerve tissues and are upregulated with neural damage [27]. Bovine LF administration has been reported to stimulate nerve growth factor secretion and aid neural healing in mice; this may explain the improvement in tear production observed in this study [27]. While reflex tear secretion is disrupted by aging [28]–[30], LF may protect nerves to maintain lacrimal gland secretory function.

Our real-time PCR investigation revealed the suppression of lacrimal gland inflammation. In vivo kinetics of LF has not been clarified completely. However, LF is known as remarkably resistant to proteolytic degradation including trypsin and chymotrypsin [31]. and when LF solution was administrated, immunoreactive LF was able to be detected in various tissues including mesenteric fat tissue, liver, renal and brain [32], [33]. furthermore, recent investigation demonstrated LF is transported from the small intestine into the lymph ducts and reaches tissues via lymphatic pathway [25], [32], [33]. Thus, since we believe that orally administered LF may increase the concentration of LF in the ocular surface, we examined the RNA expression for conjunctival inflammatory cytokines. Indeed, the expression of inflammation-related genes (MCP-1 and TNF-α) decreased in the conjunctivae of the LF-treated group (Figures 3 and 5). LF can directly inhibit the production of several cytokines, including TNF-α and interleukin 1β, via receptor-mediated signaling pathways [4], [5]. For example, in rat adjuvant-induced arthritis, LF suppresses inflammation by downregulating TNF-α and upregulating interleukin 10 [5].

Several previous reports have revealed that aging induces aggregation of inflammatory cells in lacrimal glands [18], [34]. LF prevents inflammatory cell infiltration, which may be related to oxidative stress, in aged mice. We previously reported that calorie restriction prevents decline in lacrimal gland function and morphological changes in middle-aged rats, which may also be associated with the reduction in oxidative stress and inflammation [18].Aging has been recognized, in part, as a result of accumulation of oxidative stress, generated continuously during the course of metabolic processes. Thus, the observed reduction in oxidative stress markers in the serum of the LF-treated group suggests that LF has similar action to calorie restriction. Furthermore, recently, our research group revealed that ROS induced lacrimal gland dysfunction using Superoxide dismutase 2 knockout mice and smoking rats [35]. It is plausible that LF preserves lacrimal functions by attenuating oxidative damage in the lacrimal glands, which is further supported by previously documented anti-oxidative effects of LF in the liver [6].

In accord with previous studies [36], [37], we observed that the non-esterified fatty acid levels decreased significantly in the LF-treated group. Triglyceride levels showed a similar tendency, although no marked differences could be found. Furthermore, we observed a significant LF-induced LDL-C decrease. Since LDL-C is recognized as the major determinant of endothelial dysfunction and oxidative stress in patients with coronary artery disease [38], our findings might augment the clinical benefits of intensive lipid-lowering therapy [39].

Furthermore, we detected LF-related decline in unsaturated and total iron-binding capacity of the serum, which is in agreement with the iron-binding properties of LF, acting as an iron chelator [40]. Recently, it has been shown that iron accumulation induces oxidative stress and that iron overload increases the risk of cardiovascular diseases [41] Moreover, an oral iron chelator has been reported to prevent iron overload-induced retinal degeneration [42]. LF is capable of binding 2 atoms of iron, implicated in the formation of free radicals [43]; thus, the chelating properties of LF could further boost its therapeutic potential in reducing oxidative stress [44], [45]. In the present study, we used enteric-coated LF, which is more efficiently absorbed in the intestine and has superior anti-oxidative efficacy, as compared to plain LF [46]. It has been reported that apo-LF may be even more effective in oxidative stress reduction, as it exhibits stronger iron-chelating properties [44]. Therefore, further investigation of the superior anti-oxidative characteristics of apo-LF is needed.

Lacrimal acinar cells synthesize and secrete proteins into the tear fluid [47], [48]. These include lysozyme, LF, lipocalin, and secretory IgA. While diminished lipocalin secretion has been found in patients with seborrheic blepharitis and meibomian gland dysfunction, a decrease in LF discharge has been associated with Sjögren and Stevens-Johnson syndromes [49], [50]. Tear proteins themselves can act as regulators of tear secretion [51]. Thus, LF, in addition to reducing oxidative stress, may improve tear secretion directly. Further investigations are needed to clarify this assumption.

Further, as described above, LF can directly inhibit the production of several inflammatory-related cytokines [4], [5], increase goblet cells [23], [24], therefore, not only administration but the effects of topical LF, and apo-LF which have a superior anti-oxidative characteristics are needed.

In summary, LF may attenuate oxidative stress damage and suppress inflammatory mediators in the lacrimal glands, as well as preserve the lacrimal gland function. Although the detailed mechanisms remain to be clarified, we believe that the effect of LF on tear secretion is a combination of its direct impact on the lacrimal glands and the overall improvement in body metabolism. Thus, the use of LF as a nutritional supplement may be a novel and safe treatment alternative for patients with dry eye syndrome. It could also prove beneficial in the therapy of other age-related diseases. To date, we have focused on highly specific, topical treatment options for dry eye diseases, namely eye drops. Now, we believe it is crucial to embrace a more universal approach for those diseases such as LF supplementation as this study demonstrates.
